# Functional Residues in Proteins

**DOI:** 10.4061/2011/606354

**Published:** 2011-11-03

**Authors:** Shandar Ahmad, Jung-Ying Wang, Zulfiqar Ahmad, Faizan Ahmad

**Affiliations:** ^1^National Institute of Biomedical Innovation, Osaka, Japan; ^2^Lunghwa University of Science and Technology, Taiwan; ^3^Department of Biology, Alabama A&M University, Normal, AL 35762, USA; ^4^Centre for Interdisciplinary Research in Basic Sciences, India

We are delighted to present a special issue on the subject of functional residues of proteins. Finding focused articles within the time frame of a special issue is always challenging, which is further compounded by authors' reluctance in making their contributions to new journals. We have been fortunate enough that some leading researchers in the field agreed to support this special issue and contributed their work at our request. We present here nine articles on various aspects of the subject. We received some more submissions, which could not be accommodated because of a strict quality control, we tried to maintain. Finally compiled special issue starts with a very interesting commentary on posttranslated modifications leading to normal and disease-associated biological functions, contributed by T. M. Karve and A. K. Cheema. This is followed by the analysis of a specific biological system of ATP synthetase, contributed by Z. Ahmad et al., exploring its catalytic sites and focusing on charged residues. R. Prasad et al. then describe a related system of ABC transporters and review the state of the knowledge on its functional residues. Functional residues in a protein do not always follow a universal pattern, and this is demonstrated by R. Kumar et al. in describing the dynamic structure of estrogen receptors. This paper is then followed by a special group of functional residues, that is, cysteins in sulfurtransferases by N. Nagahara. Viola et al. present an excellent study of catalytic machinery in the biosynthesis of amino acids. Subsequntly, K. M. Fukasawa et al. then present their analysis of functionally relevant zinc-binding sites in metalloproteases. Subsequently, M. S. Khan et al. provide an elaborate discussion on the mechanism of serpin inhibition, specially focusing on the role of polymerization. Finally, M. A. Kabir et al. present an investigation of a eukaryotic chaperon, involved in protein-folding.

Thus, the special issue covers a wide range of systems and describes an overview of some of the most significant studies on the subject of finding functional residues in proteins. We would like to express our most sincere gratitude to the authors who agreed to contribute and referees who provided useful feedback, allowing the papers to meet the high standards of publication.

We hope that the readers of Journal of Amino Acids will find these articles of great interest and look forward to any comments to improve our future efforts.

*Shandar Ahmad* *Shandar Ahmad* *Jung-Ying Wang* *Jung-Ying Wang* *Zulfiqar Ahmad* *Zulfiqar Ahmad* *Faizan Ahmad* *Faizan Ahmad*

